# *Plasmodium falciparum* HRP2 ELISA for analysis of dried blood spot samples in rural Zambia

**DOI:** 10.1186/s12936-017-1996-4

**Published:** 2017-08-23

**Authors:** Lauren E. Gibson, Christine F. Markwalter, Danielle W. Kimmel, Lwiindi Mudenda, Saidon Mbambara, Philip E. Thuma, David W. Wright

**Affiliations:** 1 Department of Chemistry, Vanderbilt University, Nashville, TN 37235 USA; 2 Department of Chemistry and Biochemistry, Present Address: Elizabethtown College, Elizabethtown, PA 17022 USA; 3 Macha Research Trust, Choma, Zambia

**Keywords:** Dried blood spots, ELISA, Malaria, *Plasmodium falciparum* histidine-rich protein 2, Biomarker clearance

## Abstract

**Background:**

Dried blood spots are commonly used for sample collection in clinical and non-clinical settings. This method is simple, and biomolecules in the samples remain stable for months at room temperature. In the field, blood samples for the study and diagnosis of malaria are often collected on dried blood spot cards, so development of a biomarker extraction and analysis method is needed.

**Methods:**

A simple extraction procedure for the malarial biomarker *Plasmodium falciparum* histidine-rich protein 2 (HRP2) from dried blood spots was optimized to achieve maximum extraction efficiency. This method was used to assess the stability of HRP2 in dried blood spots. Furthermore, 328 patient samples made available from rural Zambia were analysed for HRP2 using the developed method. These samples were collected at the initial administration of artemisinin-based combination therapy and at several points following treatment.

**Results:**

An average extraction efficiency of 70% HRP2 with a low picomolar detection limit was achieved. In specific storage conditions HRP2 was found to be stable in dried blood spots for at least 6 months. Analysis of patient samples showed the method to have a sensitivity of 94% and a specificity of 89% when compared with microscopy, and trends in HRP2 clearance after treatment were observed.

**Conclusions:**

The dried blood spot ELISA for HRP2 was found to be sensitive, specific and accurate. The method was effectively used to assess biomarker clearance characteristics in patient samples, which prove it to be ideal for gaining further insight into the disease and epidemiological applications.

**Electronic supplementary material:**

The online version of this article (doi:10.1186/s12936-017-1996-4) contains supplementary material, which is available to authorized users.

## Background

Sample collection is a major challenge for disease diagnosis in non-clinical settings. In low-resource areas, the collection of venous blood is not always feasible, so blood samples are often collected via finger prick and stored on filter paper cards. These samples, called dried blood spots (DBS), result in increased biomolecule sample stability compared to liquid samples, as they can be stored in ambient conditions. Additionally, DBS samples have a low biohazard risk and thus can be easily shipped [1, 2]. These advantages have led to the commercial availability of several types of DBS collection cards with different features including easy labelling and incorporation of reagents for blood lysis or biomarker stability [3].

Dried blood spot samples have proven useful for the analysis of a variety of markers including proteins, nucleic acids, and drugs. Methods employing DBS require an extraction procedure followed by techniques such as ELISA or PCR to analyse the extracted samples [4]. For example, detection of hepatitis C, a measles specific antibody, and *Streptococcus pneumoniae* from DBS samples were shown to be reliable when validated against gold standards for detection [5–7]. Additionally, dried blood spots are extensively used in newborn screening programmes which test for congenital disorders including those that are metabolic, such as hyperthyroidism, and those that are genetic, such as cystic fibrosis [8–10].

Malaria, a parasitic disease transmitted through mosquitoes, caused 212 million infections in 2015. A majority of these cases occurred in Africa, where access to clinical laboratories is limited [11]. Malaria is regularly diagnosed with rapid diagnostic tests (RDTs) that detect *Plasmodium falciparum* histidine-rich protein 2 (HRP2), a uniquely structured and highly stable protein biomarker [12]. In this work, a method for the extraction of HRP2 from dried blood spots was optimized. Various parameters were explored including extraction buffer, mixing method and extraction time. Additionally, the percent recovery of HRP2 and the biomarker stability were studied. Following extraction, a previously developed HRP2 ELISA was used for biomarker quantification [13]. This ELISA can detect low-level or asymptomatic infections that may otherwise be undetectable, and its high-throughput nature is useful for epidemiological studies [14]. The coupling of the optimized extraction method with this ELISA allowed for detection of HRP2 from DBS; a procedure that was then used to test patient samples in rural Zambia. This study allowed for determination of the method sensitivity, specificity and reproducibility, as well as biomarker clearance characteristics.

## Methods

### Materials

Human Whole Blood (K3 EDTA) was purchased from Bioreclamation IVT (Catalog No. HMWBEDTA3). *Plasmodium falciparum* D6 parasite was cultured in house. Recombinant HRP2 protein (rc-HRP2) for traditional ELISA standard curve was purchased from Immunology Consultants Laboratory Inc. (Catalog No. AGPF-55). ELISA capture and detection antibodies were acquired from Abcam Inc. (Catalog Nos. ab9206 and ab30384). Immulon 2HB ELISA plates (Catalog No. 14-245-61) and TMB One ELISA substrate (Catalog No. PR-G7431) were purchased from Fisher Scientific. 903 Protein Saver cards and Whatman 3 filter paper, were purchased from GE Healthcare Life Sciences (Catalog Nos. 10534612 and 1003-055). Biopunches, 6 mm, were acquired from Ted Pella Inc. (Catalog No. 15111-60). All other reagents were purchased from either Fisher Scientific or Sigma Aldrich. DBS extraction was performed with a Fisher Scientific Analog Vortex Mixer (Catalog No. 02-215-365). Absorbance measurements were collected on a Biotek Synergy H4 microplate reader (Vanderbilt University) or Biotek ELx808 microplate reader (Macha Research Trust).

### *Plasmodium falciparum* histidine-rich protein 2 ELISA protocol

The HRP2 ELISA protocol has been previously reported [13]. Briefly, 1 µg/mL of anti-HRP2 IgM (ab9206) in PBS was immobilized for 1 h in an Immulon 96-well plate, which was then blocked with 5% BSA in PBST (1× PBS, 0.1% Tween-20) for 2 h. Samples were then diluted in sample buffer (PBST, 0.1% BSA) and added to the plate for 2 h. Finally, horseradish peroxidase conjugated detection antibody (ab30384) was added at 0.5 µg/mL in PBST with 0.5% BSA for 1 h. Signal was visualized with TMB-One, a commercially available 3,3′,5,5′-tetramethylbenzidine (TMB) substrate and stopped with 2 M sulfuric acid after 10 min. Absorbance was read at 450 nm.

### Dried blood spot extraction

DBS were prepared by spiking *P. falciparum* D6 parasite culture into whole blood and spotting 10 μL onto Whatman 903 Protein Saver cards (903 cards) or Whatman 3 filter paper (W3). Extraction was performed on 6 mm spots. Two spots were extracted for each sample, in one, 2-mL microcentrifuge tube with 300 µL PBST extraction buffer. The tubes were vortexed at maximum speed (3200 rpm) for 10 min. Afterwards, tubes were centrifuged to remove bubbles and spin down the DBS paper. The supernatant was then removed and analysed by ELISA.

### Dried blood spot *Plasmodium falciparum* histidine-rich protein 2 ELISA

The previously reported HRP2 ELISA [13] was performed with the following modifications: (1) The standard curve was made from DBS standards to incorporate extraction conditions into the standard curve, (2) samples were directly added to the plate in the PBST extraction buffer instead of being diluted in sample buffer.

### Percent recovery calculation

Percent recovery was determined by comparing ELISA signal from a blank (whole blood diluted 1:14 in PBST) and a positive control (whole blood spiked with parasite to 200 parasites/µL, diluted 1:14 in PBST) to ELISA signal from an extracted DBS sample. For example, percent recovery for a 200 parasites/µL DBS sample was calculated using the following equation:

$$\% {\text{ Recovery HRP2}} = \left( {\frac{{A_{DBS} - A_{blank} }}{{A_{positive} - A_{blank} }}} \right) \times 100$$

### Stability study

Dried blood spots were prepared on 903 cards and W3. The spots were stored in Ziploc bags containing desiccant at the following temperatures: room temperature (RT), 4, −20 and −80 °C. Samples were stored for various lengths of time, extending up to 6 months, and analysed for percent recovery of HRP2.

### Study setting

Patient samples were collected during a separate study carried out in the Nchelenge District of Zambia, an area where malaria transmission has been sustained at a high level despite interventions [15]. The original study was designed to determine parasite clearance rates after treatment with artemisinin-based combination therapy (ACT) in children under 5 years of age presenting with uncomplicated malaria to the local clinic. The anonymized samples were made available for the current study to determine if HRP2 clearance could be used as a proxy for parasite clearance rates.

### Patient recruitment and ethics

Children who arrived at the clinic and tested positive for malaria according to SD Bioline HRP2 malaria RDTs were recruited for this study after the parent or guardian provided signed informed consent. The study and samples were collected under IRB approval TDRC/C4/09/2014 as well as after approval was granted by the Zambian National Health Research Authority (MH/101/17/6).

### Patient samples

Patient finger-prick blood samples were collected on 903 cards between December 2014 and August 2015. DBS HRP2 ELISA analysis was done in July 2016. In the interim between collection and analysis, the DBS samples were stored at −20 °C. At the time of collection, finger-prick samples were analysed by thick smear microscopy to determine quantitative parasite levels by counting parasites per 200 white blood cells and then using an estimate of 8000 WBC/μL to obtain the parasite count for that particular time point. This analysis was used as the gold standard in this study. After diagnosis by RDT and *P. falciparum* parasitaemia confirmation by a malaria smear, patients were enrolled and treated with artemether–lumefantrine (Coartem®). Thick blood film malaria smears and DBS samples were collected at 15 time points: 0, 6, 12, 18, 24, 30, 36, 42, and 48 h and days 3, 7, 14, 21, 28, and 35. In the current study, samples from time points 0, 6, 12 and 18 h as well as days 28 and/or 35 were made available and analysed for 35 patients. Additionally, all 15 time points were studied for 10 patients.

### Patient sample dried blood spot ELISA

Before analysis, patient samples were coded to ensure assays were performed blinded to microscopy results. To begin, one, 6-mm DBS spot was placed in a 2 mL microcentrifuge tube with 300 µL of PBST extraction buffer. After vortexing for 10 min, the supernatants were removed for analysis by HRP2 ELISA with two modifications: (1) the standard curve (0–20 parasites/µL) was made from D6 parasite culture spiked into 1:29 (v:v) whole blood in PBST to approximate DBS extract matrix, and (2) after extraction, the samples were diluted tenfold in PBST before addition to the plate.

### Sample size calculation

Appropriate sample size is essential for validation of diagnostic tests. Buderer reported an equation for calculation of sample size to ensure that a planned study would have sufficient power [16, 17]. This number is dependent on estimated sensitivity or specificity, and equations for each are shown below. To ensure sufficient sample size, the larger of the two was chosen.

$$n_{Se} = \frac{{Z_{{\frac{\alpha }{2}}}^{2} Se\left( {1 - Se} \right)}}{{d^{2} \times Prev}}\;\;\;\;\;\;\;n_{Sp} = \frac{{Z_{{\frac{\alpha }{2}}}^{2} Sp\left( {1 - Sp} \right)}}{{d^{2} \times \left( {1 - Prev} \right)}}$$

Variables are defined as: n = number of samples, Se = sensitivity, Sp = specificity, Z = Z score (1.96 for this analysis), d = confidence interval, and Prev = prevalence of disease. For this study, we chose d = 0.1. For this analysis, the prevalence was approximated at 70% and the desired sensitivity and specificity were 90 and 80% respectively. When these values were incorporated into the above equation, *n*_*Se*_ = 49 samples and *n*_*Sp*_ = 205 samples. A sufficient sample size of 238 DBS was analysed.

### Data analysis

For DBS samples, the concentration of HRP2 extracted was determined via standard curves. For the standard D6 parasite culture, 1 parasite/µL = 1.7 pM HRP2 (see Additional file 1: Figure S1). All error bars shown in the data represent the standard deviation between replicates of the samples analysed. Intra-assay variability was calculated by finding the standard deviation of replicates run at a single concentration, in a single assay, and dividing by the mean of the replicates. Inter-assay variability was found by determining the standard deviation of a single concentration for assays run on different days and dividing by the mean value for that concentration. The sensitivity and specificity of the DBS HRP2 ELISA method was determined through a ROC curve analysis using microscopy results as the gold standard.

## Results

### Optimization of extraction parameters

**Fig. 1:**
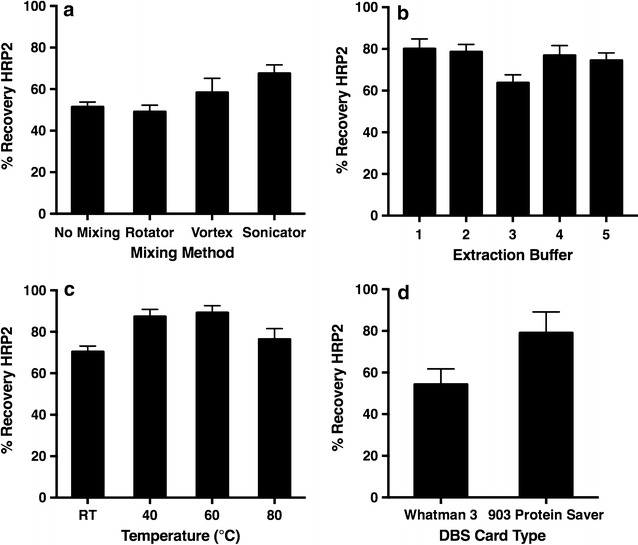
Optimization of parameters for the extraction of *Plasmodium falciparum* histidine-rich protein 2 (HRP2) from dried blood spot (DBS) samples. **a** Mixing method, **b** extraction buffer, **c** temperature and **d** dried blood spot card type

In order to analyse HRP2 in a DBS sample, the extraction method had to be optimized. The technique used for mixing was chosen by extraction into PBST using rotisserie, vortex, and sonicator mixing as well as without mixing. It was found that recovery of HRP2 increased as the energy imparted by the mixing method increased (Fig. 1a). Vortex mixing was selected as it resulted in a high recovery and is also feasible in low resource settings [18]. No difference in recovery was seen between 10 and 30 min of mixing (see Additional file 1: Figure S2).

Extraction buffer compositions with varying salt concentrations and lysis reagents were also explored. The composition of the buffers analysed are listed in Additional file 1: Table S1. A greater percent recovery of HRP2 was observed with buffers which contained nonionic surfactants, likely due to greater solubilization of the protein by the long polyethylene glycol chains. Salt concentration did not affect recovery (Fig. 1b). PBST (Buffer 1) was chosen as the extraction buffer.

The ratio of extraction buffer to 6 mm DBS was also examined for impact on HRP2 recovery. Our study showed no significant difference in recovery for volumes of 150–250 µL per 6 mm DBS (see Additional file 1: Figure S3). Similarly it was found that extracting two, 6 mm DBS in 300 µL of buffer compared to one, 6 mm DBS doubled the slope of the standard curve, increasing assay sensitivity (see Additional file 1: Figure S4).

Finally, the effect of heating was analysed by placing DBS in extraction buffer and then in a heat block at 40, 60 and 80 °C for 10 min. The samples were then vortexed for 10 min before analysis by ELISA. Heating at 60 °C increased recovery of HRP2 from approximately 70% to 90% (Fig. 1c). For this method, room temperature was chosen to reduce necessary instrumentation, but a heating step can be added if increased assay sensitivity is required.

### Dried blood spot card type and *Plasmodium falciparum* histidine-rich protein 2 recovery and stability

**Fig. 2:**
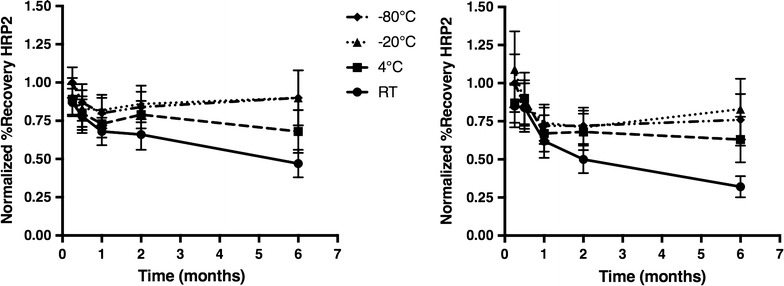
The stability of *Plasmodium falciparum* histidine-rich protein 2 (HRP2) in dried blood spots, over time, in different storage conditions. Stability on 903 Protein Saver cards (*left*) and Whatman 3 filter paper (*right*)

903 cards are designed for DBS collection while W3 is made for filtration. Because 903 cards are more expensive than W3, many field studies with limited budgets have employed W3. To determine if the paper type has an effect on HRP2 recovery, DBS samples were prepared on each card type. It was found that recovery of HRP2 from 903 cards was consistently 20% greater than recovery from samples on W3 (Fig. 1d). HRP2 stability was also assessed with both card types. It was observed that HRP2 was less stable in W3 samples with only 30% of the initial HRP2 recovery observed after being stored at room temperature for 6 months. Samples on 903 cards retained 50% of the initial recovery after being stored for 6 months at room temperature. Additionally, samples on 903 cards retained 100% of the original recovery after storage for 6 months at −20 and −80 °C (Fig. 2).

### Comparison to traditional *Plasmodium falciparum* histidine-rich protein 2 ELISA

**Fig. 3:**
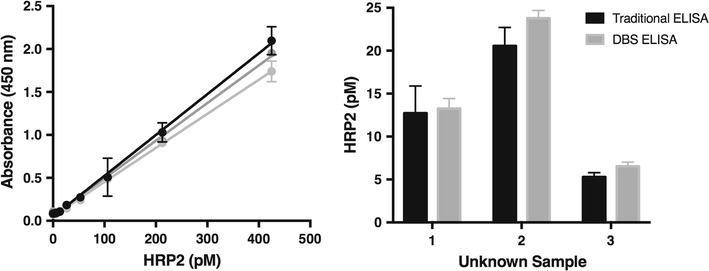
The reproducibility and accuracy of the developed dried blood spot (DBS) *Plasmodium falciparum* histidine-rich protein 2 (HRP2) ELISA. DBS standard curves run on three different days (*left*) and comparison of HRP2 values between the DBS ELISA and a traditional ELISA (*right*)

Using the optimized protocol for extraction of two, 6 mm DBS in 300 µL of PBST, with vortex mixing for 10 min, standard curves were generated from parasite-spiked DBS samples on 903 cards. The resulting curves were highly reproducible with an intra-assay variability of 7% and inter-assay variability of 10% (n = 3) (Fig. 3). The limit of detection (LOD), defined as 3σ_blank_/slope, was 4 ± 5 parasites/µL (8 ± 8 pM HRP2). Samples were then analysed simultaneously on the previously reported HRP2 ELISA and the DBS HRP2 ELISA [13]. Upon analysis with a paired t test, all p values were greater than 0.05 proving there was no statistical difference between the HRP2 levels found by the two methods (Fig. 3).

### Analysis of patient samples from rural Zambia

**Fig. 4:**
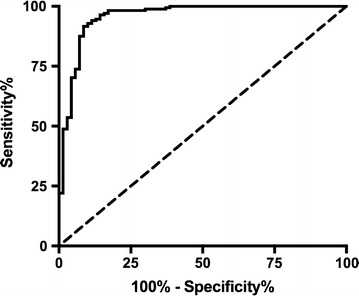
ROC curve for dried blood spot (DBS) *Plasmodium falciparum* histidine-rich protein 2 (HRP2) ELISA when compared to clinical microscopy values for patient samples from rural Zambia. Sensitivity of the assay was 94% (CI 89–97%) and specificity was 89% (CI 79–95%) when the threshold was set at 3.5 pM HRP2

The standard curves used for analysis of DBS patient samples were made from parasite-spiked diluted whole blood. These assays, performed at Macha Research Trust, had an intra-assay variability of 6% and an inter-assay variability of 18% (n = 14). The LOD determined by 3σ_blank_/slope was 0.165 ± 0.003 pM HRP2 (see Additional file 1: Figure S5). 238 DBS patient samples collected within 24 h or at least 28 days after malaria diagnosis and treatment were analysed by DBS HRP2 ELISA (see Additional file 1: Table S2). Using microscopy as the gold standard, a ROC curve was used to determine the sensitivity and specificity of the assay. It was found to have sensitivity of 94% (CI 89–97%) and specificity of 89% (CI 79–95%) when the threshold was set at 3.5 pM HRP2 (Fig. 4). The area under the ROC curve was 0.96, indicating excellent accuracy of the method. Furthermore, using this threshold, the positive predictive value (PPV) for the assay was 95% and the negative predictive value (NPV) was 86%.

### *Plasmodium falciparum* histidine-rich protein 2 clearance

**Fig. 5:**
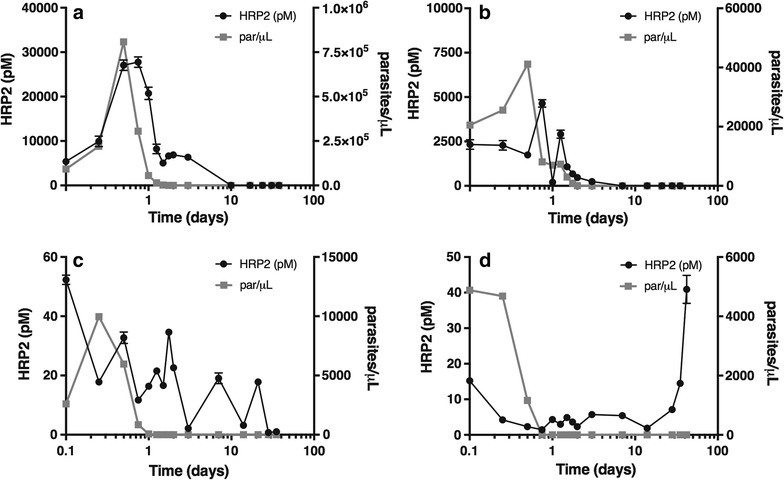
Representative data of different *Plasmodium falciparum* histidine-rich protein 2 (HRP2) clearance trends that were observed in patient samples collected at 15 time points, over a 35-day period. The concentration of HRP2 extracted from the dried blood spot (DBS) samples and the parasitaemia determined by microscopy are plotted against time. **a** Patient 27, **b** Patient 50, **c** Patient 43 and **d** Patient 44

For 10 patients, all 15 time points were analysed and used to study the clearance of parasite and HRP2 after treatment (Fig. 5; Additional file 1: Figure S6). For these ten patients, the median clearance time of the parasite was 1.38 days (0.5–3 days). For HRP2, two patients were HRP2 negative by the ELISA at all time points, so they were excluded from the median calculation. The median clearance time of HRP2 for the remaining eight patients was 5 days (0.25–21 days). Various HRP2 clearance trends were observed when looking at each patient individually. As mentioned previously, two patients with low initial parasitaemias (see Additional file 1: Figure S6C, F), were negative for HRP2 according to the 3.5 pM threshold. Patient 51 (see Additional file 1: Figure S6E) had relatively low HRP2 levels before a large spike at t = 1 day. Patients 25, 27 and 45 (Fig. 5a; Additional file 1: Figure S6A, B, respectively) all showed HRP2 levels which corresponded with parasite load but with a lag in clearance time. Patients 49 (see Additional file 1: Figure S6D) and 50 (Fig. 5b) displayed the same trend except for a couple of spikes corresponding to a decrease in parasite levels. The HRP2 level in Patient 43 (Fig. 5c) spiked and fell throughout the study but was cleared by the last time point. Finally, Patient 44 (Fig. 5d) showed decreasing HRP2 levels over time until they began to rise at the last three time points while parasite levels remained negative.

**Fig. 6:**
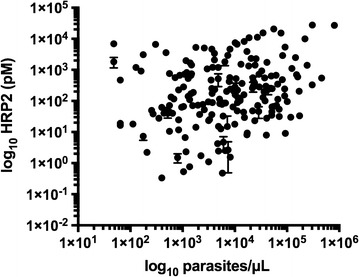
Correlation between parasite and HRP2 levels that were observed in the 328 patient samples from rural Zambia. Spearman correlation coefficient (ρ) = 0.70 (95% CI 0.63–0.75, p < 0.0001)

To ascertain whether there was a correlation between parasite and HRP2 levels, the DBS HRP2 ELISA results for all 328 patient samples were compared to microscopy. A positive, linear association was observed, with a Spearman correlation coefficient (ρ) equal to 0.70 (95% CI 0.63–0.75, p < 0.0001) (Fig. 6).

## Discussion

### *Plasmodium falciparum* histidine-rich protein 2 and dried blood spots

Extraction of HRP2 from DBS was achieved reproducibly, but several factors affected its efficiency, including the type of paper on which the DBS was prepared. This is an important variable to consider, as 903 cards cost up to eight times more than W3. As shown in the results section, 903 cards had up to a 20% greater recovery of HRP2, and HRP2 did not degrade in these cards when stored frozen for 6 months (Figs. 1d, 2). These results indicate that although the more cost effective alternative is often chosen in field study design, in this case the additional cost enhances performance. The authors conclude that 903 cards most likely provide a more stable matrix for protein storage, and their increased thickness allows for a greater volume of blood in a specific DBS area resulting in greater protein recovery.

### ROC curve analysis

ROC curves are used as a metric to determine the sensitivity and specificity of a diagnostic assay as compared to the chosen gold standard. An ideal ROC curve forms a 90º angle in the left corner of the graph, which corresponds to a test with 100% sensitivity and 100% specificity. In this analysis, the DBS HRP2 ELISA was found to have a sensitivity of 94% and a specificity of 89% with a threshold of 3.5 pM HRP2 (Fig. 4). This result demonstrates the developed DBS HRP2 ELISA is a reliable diagnostic for malaria. Reduced specificity compared to sensitivity can be attributed to the fact that the gold standard for malaria diagnosis (microscopy) detects the malaria parasite rather than the protein biomarker detected with the DBS HRP2 ELISA. This discrepancy can result in false positives by DBS ELISA, because HRP2 can persist in the blood stream days to weeks after parasite clearance [19, 20]. Furthermore, microscopy has shortcomings of its own, including a lack of sensitivity. In general, ELISA is more sensitive than microscopy [21]. As a result, false positives could result from the ability of the DBS HRP2 ELISA to detect malaria infections that are not seen by microscopy.

While a lower threshold would allow for detection of more truly HRP2-positive samples, it would also result in detection of residual HRP2 that is not the result of an active malaria infection. This observation points to a disadvantage of HRP2 as a biomarker, as its persistence can result in misdiagnosis of patients and improper treatment [22]. To address this issue, an alternative protein biomarker that quickly clears from the bloodstream, *Plasmodium* lactate dehydrogenase (*p*LDH), is being further explored in our lab [20, 23]. Nonetheless, the importance of detection of HRP2 remains, as it continues to be the most sensitive and prevalently used protein biomarker for detection of *falciparum* malaria infections [12].

### Biomarker clearance

Several different trends were observed in the clearance patterns of HRP2, indicating that the response of each patient to the disease is different. Despite this, some similarities were observed, including delayed HRP2 clearance compared to parasite clearance, a result of the persistence of HRP2 [24]. Additionally, the spikes seen in several patients directly after a decrease in parasite load are likely caused by the release of HRP2 upon parasite death [25]. Finally, in one patient, HRP2 levels increased in the final time points indicating reinfection or recrudescence may have occurred (Fig. 5d). Reinfection is when an individual is successfully treated for malaria but then becomes sick again with an entirely new malaria infection [26]. Recrudescence occurs when the same malaria infection is never fully eliminated by treatment and reappears [27]. Without sample genotyping the cause of the HRP2 spike in this sample cannot be confirmed [28, 29].

Using the ROC curve threshold, information can be gathered about infection levels and HRP2 persistence in the blood. For this study, infections levels were classified as follows: low (0–14,999 parasites/µL), medium (15,000–75,000 parasites/µL) and high (≥75,000 parasites/µL). Besides the case discussed above, Patients 3 and 37 (see Additional file 1: Table S2) had HRP2 levels that were positive at the end of the time study meaning these samples contained HRP2 as long as 30 days after the beginning of treatment. Furthermore, samples from Patients 43 and 45 (Fig. 5c; Additional file 1: Figure S6B, respectively), which were analysed for the full time study, retained HRP2 past the median time-to-clearance of 5 days. It could be expected that these extended clearance times were a result of high level infections but instead it was found that the initial parasite level for each of these patients was below 25,000 parasites/µL. This result suggests that persistence of HRP2 was a result of chronic infections. In chronic infections, low-level parasitaemias can persist in a patient for years, with minimal symptoms, and then become symptomatic [30–32]. The longevity of the infection can allow for build-up of HRP2 over time, so even though the parasite load is low, a significant amount of HRP2 persists in the blood.

### Association between parasite and biomarker levels

A correlation between parasite and HRP2 levels measured by microscopy and DBS HRP2 ELISA respectively was observed (Fig. 6). The Spearman correlation coefficient (ρ) was 0.70, and this result statistically proves an association between these two variables. The strength of the association is considered to be moderate (ρ = 0.5–0.7) to high (ρ = 0.7–0.9) [33]. As can be seen in Additional file 1: Figure S1, in the case of parasite culture this correlation between HRP2 and parasite levels is direct. Several different factors could have contributed to the decrease in the strength of association observed with patient samples. For example, the lack of strictly controlled storage conditions for the DBS samples likely caused protein degradation and variability in extraction efficiency. As a result, in the analysis of the patient samples there were other influences on protein recovery besides the initial parasitaemia of the sample. Additionally, the amount of HRP2 produced by the parasite is dependent on the life stage and strain [25, 34]. Patient samples have much greater variety in these parameters than parasite culture, resulting in a weaker association between HRP2 and parasite levels. Other literature reports have seen similar discrepancies in patient samples, as some report correlation between HRP2 levels and disease severity, while others do not [35, 36]. Nonetheless, in this work a positive association between HRP2 and parasite levels was observed, indicating the usefulness of the HRP2 biomarker in malaria diagnosis.

## Conclusions

Dried blood spots represent a common and simple sample collection method for clinical and non-clinical settings. Samples stored in this way are stable and easily shipped. In order to analyse these samples, methods must be optimized for extraction of a specific biomarker and subsequent analysis. This is a report of a highly reproducible procedure for extraction of the malarial biomarker HRP2 and subsequent analysis of the protein by ELISA. This assay was then used to analyse patient samples in rural Zambia and was found to have a 94% sensitivity and 89% specificity. Examination of patient samples also revealed trends in biomarker clearance, showing sustained HRP2 in the blood. Furthermore, a positive, linear association between parasite and HRP2 levels was observed. Thus, the reported method is reliable for detection of malaria from commonly collected dried blood spot samples and can be applied further in epidemiological studies and to assist in the development of improved diagnostics through increased understanding of disease characteristics.

## Additional file

**Additional file 1.** Additional data from optimization of dried blood spot extraction. Reproducibility of the standard curves performed at Macha Research Trust. Dried blood spot ELISA results for patient samples and additional time study data. **Figure S1.** Amount of HRP2 in D6 parasite culture. **Figure S2.** Mixing time. **Table S1.** Extraction buffer compositions. **Figure S3.** Extraction buffer volume. **Figure S4.** Number of dried blood spots and assay sensitivity. **Figure S5.** Standard curves from Macha Research Trust. **Table S2.** Additional patient sample data. **Figure S6.** Additional time study data.

## Abbreviations

DBS: dried blood spot
HRP2: *Plasmodium falciparum* histidine-rich protein 2
903 cards: Whatman 903 Protein Saver cards
W3: Whatman 3 filter paper
TMB: tetramethylbenzidine
ACT: artemisinin-based combination therapy
