# Supplementary material for: Plasmodium falciparum HRP2 ELISA for analysis of dried blood spot samples in rural Zambia
Source: Malar J. 2017 Aug 23;16:350. doi: 10.1186/s12936-017-1996-4 (PMC5569460; doi:10.1186/s12936-017-1996-4)
Supplement: Supplementary file 1 — Additional file 1. Additional data from optimization of dried blood spot extraction. Reproducibility of the standard curves performed at Macha Research Trust. Dried blood spot ELISA results for patient samples and additional time study data. Figure S1. Amount of HRP2 in D6 parasite culture. Figure S2. Mixing time. Table S1. Extraction buffer compositions. Figure S3. Extraction buffer volume. Figure S4. Number of dried blood spots and assay sensitivity. Figure S5. Standard curves from Macha Research Trust. Table S2. Additional patient sample data. Figure S6. Additional time study data. [file 12936_2017_1996_MOESM1_ESM.docx]

**Supplementary Information**

***Plasmodium falciparum* HRP2 ELISA for analysis of dried blood spot samples in rural Zambia**

Gibson, Lauren E.,^1,2^ Markwalter, Christine F.,^1^ Kimmel, Danielle W.,^1^ Mudenda, Lwiindi,^1^ Mbambara, Saidon,^3^ Thuma, Philip E.,^3^ Wright, David W.^1*^

^1^Department of Chemistry, Vanderbilt University, Nashville, TN 37235, USA

^2^Present address: Department of Chemistry and Biochemistry, Elizabethtown College, Elizabethtown, PA 17022, USA

^3^Macha Research Trust, Choma, Zambia

*Corresponding Author: Tel: 615-322-2636, E-mail: david.wright@vanderbilt.edu

**Table of Contents**

Fig. S1 Amount of HRP2 in D6 parasite culture 2

Fig. S2 Mixing time 3

Table S1 Extraction buffer compositions 4

Fig. S3 Extraction buffer volume 5

Fig. S4 Number of dried blood spots and assay sensitivity 6

Fig. S5 Standard curves from Macha Research Trust 7

Table S2 Additional patient sample data 8

Fig. S6 Additional time study data 11

**Fig. S1** A recombinant histidine-rich protein 2 standard (rc-HRP2) was used to quantitate the amount of HRP2 in the D6 parasite culture (y = 1.69x). 1 parasite/μL = 1.7 pM HRP2.

**Fig. S2** The effect of vortex mixing time on *Plasmodium falciparum* histidine-rich protein 2 (HRP2) recovery. No difference was observed between time points.

| **Table S1** Extraction buffer compositions | |
| --- | --- |
| **Buffer** | **Composition** |
| 1 | 10 mM PB pH=7.4, 150 mM NaCl, 0.1% Tween-20 |
| 2 | 50 mM PB pH=8, 150 mM NaCl, 1% Triton X-100 |
| 3 | 50 mM PB pH=8, 150 mM NaCl, 0.5% sodium deoxycholate |
| 4 | 50 mM PB pH=8, 150 mM NaCl, 1% Triton X-100, 0.5% sodium deoxycholate |
| 5 | 50 mM PB pH=8, 300 mM NaCl, 1% Triton X-100 |

**Fig. S3** The effect of the volume of extraction buffer on *Plasmodium falciparum* histidine-rich protein 2 (HRP2) recovery. There was no difference observed with extraction buffer volume.

**Fig. S4** A dried blood spot (DBS) standard curve was performed with one and two DBS in 300 µL of extraction buffer. As was expected, when two DBS were used the assay had twice the sensitivity. One DBS y = 0.00134x, two DBS y = 0.00255x.

**Fig. S5** Standard curves performed at Macha Research Trust. Standards were made from parasite spiked diluted whole blood. These fourteen curves had an intra-assay variability of 6%, inter-assay variability of 18% and calculated LOD of 0.165 ± 0.003 pM *Plasmodium falciparum* histidine-rich protein 2 (HRP2).

| **Table S2** *Plasmodium falciparum* histidine-rich protein 2 (HRP2) levels found by HRP2 DBS ELISA for patient samples analysed at first and last time points. | | | | | | | | | |
| --- | --- | --- | --- | --- | --- | --- | --- | --- | --- |
| **Patient Sample ID** | **Time Point (days)** | **Microscopy (par/µL)** | **HRP2 DBS ELISA (pM)*** | **Standard deviation (pM)** | **Patient Sample ID** | **Time Point (days)** | **Microscopy (par/µL)** | **HRP2 DBS ELISA (pM)*** | **Standard deviation (pM)** |
| Patient1 | 0.1 | 242000 | 1410 | 90 | Patient 19 | 0.1 | 43440 | 230 | 70 |
|  | 0.25 | 8000 | 570 | 40 |  | 0.25 | 21880 | 48 | 6 |
|  | 0.5 | 840 | 227 | 5 |  | 0.5 | 10160 | 158 | 9 |
|  | 0.75 | 0 | 52 | 3 |  | 0.75 | 3720 | 89 | 4 |
|  | 37 | 0 | 0.12 | 0.03 |  | 35 | 0 | 1.04 | 0.06 |
| Patient 2 | 0.1 | 145600 | 2490 | 80 | Patient 20 | 0.1 | 3280 | 25 | 2 |
|  | 0.25 | 24360 | 1400 | 200 |  | 0.25 | 50858 | 35 | 2 |
|  | 0.5 | 22720 | 400 | 200 |  | 0.5 | 51556 | 64 | 4 |
|  | 0.75 | 10080 | 300 | 100 |  | 0.75 | 66551 | 114 | 7 |
|  | 38 | 0 | -0.03 | 0.03 |  | 41 | 0 | 0.01 | 0.04 |
| Patient 3 | 0.1 | 1440 | 670 | 40 | Patient 21 | 0.1 | 17040 | 8 | 1 |
|  | 0.25 | 3520 | 470 | 30 |  | 0.5 | 89875 | 145 | 7 |
|  | 0.5 | 3680 | 170 | 10 |  | 0.75 | 40157 | 8.0 | 0.6 |
|  | 0.75 | 1080 | 720 | 50 |  | 38 | 0 | 0.07 | 0.06 |
|  | 31 | 0 | -0.02 | 0.02 |  |  |  |  |  |
|  | 38 | 0 | 180 | 10 |  |  |  |  |  |
| Patient 4 | 0.1 | 1800 | 14 | 1 | Patient 23 | 0.1 | 159600 | 600 | 30 |
|  | 0.25 | 15280 | 29 | 2 |  | 0.25 | 12600 | 110 | 7 |
|  | 0.5 | 14720 | 43 | 3 |  | 0.5 | 3640 | 36 | 2 |
|  | 0.75 | 2120 | 37 | 4 |  | 0.75 | 840 | 25 | 2 |
|  | 31 | 0 | 1.0 | 0.2 |  | 44 | 0 | 0.03 | 0.05 |
|  | 38 | 0 | 1.1 | 0.1 |  |  |  |  |  |
| Patient 5 | 0.1 | 61680 | 670 | 30 | Patient 24 | 0.1 | 92960 | 9.1 | 0.6 |
|  | 0.25 | 29440 | 280 | 10 |  | 0.25 | 55136 | 66 | 3 |
|  | 0.5 | 22469 | 300 | 100 |  | 0.75 | 3160 | 23 | 4 |
|  | 0.75 | 1600 | 190 | 10 |  | 38 | 0 | 0.09 | 0.04 |
|  | 35 | 0 | 0.48 | 0.08 |  |  |  |  |  |
| Patient 6 | 0.1 | 36400 | 200 | 10 | Patient 26 | 0.1 | 3640 | 4600 | 400 |
|  | 0.25 | 51239 | 45 | 2 |  | 0.25 | 4080 | 3400 | 200 |
|  | 0.5 | 71016 | 31 | 2 |  | 0.5 | 1480 | 1400 | 100 |
|  | 0.75 | 1560 | 71 | 4 |  | 0.75 | 128 | 1200 | 200 |
|  | 40 | 0 | 0.66 | 0.03 |  | 28 | 0 | 0.13 | 0.07 |
|  |  |  |  |  |  | 35 | 0 | 0.46 | 0.07 |
| Patient 7 | 0.1 | 23720 | 380 | 17 | Patient 28 | 0.1 | 26980 | 11300 | 500 |
|  | 0.25 | 14960 | 160 | 17 |  | 0.25 | 18760 | 10900 | 400 |
|  | 0.5 | 560 | 72 | 3 |  | 0.5 | 1400 | 1700 | 200 |
|  | 0.75 | 400 | 62 | 3 |  | 0.75 | 48 | 1829.514 | 700 |
|  | 35 | 0 | 0.049 | 0.008 |  | 37 | 0 | 0.17 | 0.04 |
| Patient 8 | 0.1 | 66400 | 350 | 40 | Patient 31 | 0.1 | 10160 | 7500 | 600 |
|  | 0.25 | 124160 | 1120 | 50 |  | 0.25 | 4800 | 500 | 200 |
|  | 0.5 | 52045 | 249 | 6 |  | 0.5 | 2440 | 77 | 8 |
|  | 0.75 | 1280 | 530 | 20 |  | 0.75 | 1360 | 110 | 6 |
|  | 36 | 0 | 0.8 | 0.2 |  | 35 | 0 | 0.03 | 0.02 |
| Patient 9 | 0.1 | 90080 | 240 | 10 | Patient 32 | 0.1 | 4800 | 1200 | 50 |
|  | 0.25 | 3640 | 250 | 10 |  | 0.25 | 1200 | 800 | 40 |
|  | 0.5 | 3120 | 94 | 4 |  | 0.5 | 720 | 560 | 30 |
|  | 0.75 | 272 | 38 | 3 |  | 0.75 | 0 | 880 | 40 |
|  | 38 | 0 | 0.56 | 0.05 |  | 41 | 0 | 0.15 | 0.01 |
|  |  |  |  |  |  | 48 | 0 | 0.18 | 0.02 |
| Patient 10 | 0.1 | 29000 | 720 | 50 | Patient 33 | 0.1 | 17040 | 3900 | 200 |
|  | 0.25 | 37246 | 550 | 20 |  | 0.25 | 560 | 760 | 50 |
|  | 0.5 | 39500 | 300 | 10 |  | 0.5 | 640 | 40 | 2 |
|  | 0.75 | 480 | 51 | 2 |  | 0.75 | 520 | 40 | 10 |
|  | 39 | 0 | 0.08 | 0.04 |  | 38 | 0 | 0.15 | 0.01 |
| Patient 11 | 0.1 | 1120 | 6.7 | 0.8 | Patient 34 | 0.1 | 3760 | 1200 | 100 |
|  | 0.25 | 176 | 7 | 2 |  | 0.25 | 100652 | 100 | 20 |
|  | 0.5 | 0 | 5.4 | 0.6 |  | 0.5 | 70788 | 54 | 3 |
|  | 0.75 | 0 | 2 | 2 |  | 0.75 | 62725 | 72 | 4 |
|  | 33 | 0 | -0.06 | 0.008 |  | 44 | 0 | 0.05 | 0.01 |
|  | 40 | 0 | -0.049 | 0.008 |  |  |  |  |  |
| Patient 12 | 0.1 | 49960 | 630 | 90 | Patient 35 | 0.1 | 6440 | 1800 | 500 |
|  | 0.25 | 32836 | 930 | 40 |  | 0.25 | 4080 | 2100 | 400 |
|  | 0.5 | 8240 | 240 | 10 |  | 0.5 | 1480 | 740 | 50 |
|  | 0.75 | 1040 | 280 | 10 |  | 0.75 | 600 | 130 | 10 |
|  | 42 | 0 | -0.01 | 0.01 |  | 39 | 0 | 1.2 | 0.1 |
|  |  |  |  |  |  | 46 | 0 | 0.07 | 0.01 |
|  |  |  |  |  |  |  |  |  |  |
|  |  |  |  |  |  |  |  |  |  |
|  |  |  |  |  |  |  |  |  |  |
|  |  |  |  |  |  |  |  |  |  |
| Patient 13 | 0.1 | 13760 | 370 | 30 | Patient 36 | 0.1 | 18440 | 1330 | 60 |
|  | 0.25 | 6240 | 86 | 7 |  | 0.25 | 1160 | 2000 | 100 |
|  | 0.5 | 64 | 19.0 | 0.8 |  | 0.5 | 480 | 3600 | 200 |
|  | 0.75 | 0 | 8 | 2 |  | 0.75 | 176 | 3100 | 200 |
|  | 41 | 0 | -0.04 | 0.01 |  | 41 | 0 | 0 |  |
| Patient 14 | 0.1 | 5160 | 82 | 4 | Patient 37 | 0.1 | 13120 | 1520 | 60 |
|  | 0.25 | 173667 | 33 | 3 |  | 0.25 | 5080 | 115 | 2 |
|  | 0.5 | 170667 | 200 | 20 |  | 0.5 | 1960 | 73 | 1 |
|  | 0.75 | 28629 | 50 | 20 |  | 0.75 | 680 | 117 | 2 |
|  | 38 | 0 | -0.06 | 0.04 |  | 35 | 0 | 117 | 8 |
| Patient 15 | 0.1 | 460200 | 540 | 30 | Patient 38 | 0.1 | 82320 | 2900 | 100 |
|  | 0.25 | 304000 | 330 | 10 |  | 0.25 | 7840 | 420 | 50 |
|  | 0.5 | 152000 | 700 | 70 |  | 35 | 0 | 2.6 | 0.1 |
|  | 0.75 | 4120 | 230 | 10 |  |  |  |  |  |
|  | 40 | 0 | 0.4 | 0.1 |  |  |  |  |  |
| Patient 16 | 0.1 | 81000 | 480 | 50 | Patient 39 | 0.1 | 10560 | 520 | 40 |
|  | 0.25 | 9240 | 64 | 7 |  | 0.25 | 21788 | 261 | 7 |
|  | 0.5 | 17240 | 9.3 | 0.3 |  | 0.5 | 11240 | 90 | 10 |
|  | 0.75 | 256 | 49 | 3 |  | 0.75 | 5720 | 78 | 3 |
|  | 39 | 0 | 0.60 | 0.06 |  | 35 | 0 | 2.0 | 0.1 |
| Patient 17 | 0.1 | 7200 | 10 | 20 | Patient 40 | 0.1 | 22120 | 560 | 20 |
|  | 0.25 | 117819 | 30 | 3 |  | 0.25 | 12640 | 104 | 4 |
|  | 0.5 | 1280 | 140 | 10 |  | 0.5 | 11480 | 129 | 6 |
|  | 0.75 | 5400 | 13 | 1 |  | 0.75 | 960 | 63 | 3 |
|  | 39 | 0 | 0.3 | 0.2 |  | 35 | 0 | 2.2 | 0.1 |
| Patient 18 | 0.1 | 6640 | 86 | 8 |  |  |  |  |  |
|  | 0.25 | 440 | 40 | 2 |  |  |  |  |  |
|  | 0.5 | 352 | 28 | 1 |  |  |  |  |  |
|  | 0.75 | 112 | 18 | 3 |  |  |  |  |  |
|  | 31 | 0 | 0.06 | 0.04 |  |  |  |  |  |
|  | 39 | 0 | 0.2 | 0.2 |  |  |  |  |  |
| * Values were not corrected for inherent dilution that occurs upon DBS extraction. | | | | | | | | | |

**Fig. S6** Clearance trends of both parasite and *Plasmodium falciparum* histidine-rich protein 2 (HRP2) over 15 time points for A) Patient 25, B) Patient 45, C) Patient 46, D) Patient 49, E) Patient 51 and F) Patient 53.
